# An Advanced Bio-Inspired PhotoPlethysmoGraphy (PPG) and ECG Pattern Recognition System for Medical Assessment

**DOI:** 10.3390/s18020405

**Published:** 2018-01-30

**Authors:** Francesco Rundo, Sabrina Conoci, Alessandro Ortis, Sebastiano Battiato

**Affiliations:** 1 STMicroelectronics—ADG Central R&D, 95121 Catania, Italy; sabrina.conoci@st.com; 2 Department of Mathematics and Computer Science, University of Catania, 95125 Catania, Italy; ortis@dmi.unict.it (A.O.); battiato@dmi.unict.it (S.B.)

**Keywords:** PPG, ECG, pattern recognition, physiological signal

## Abstract

Physiological signals are widely used to perform medical assessment for monitoring an extensive range of pathologies, usually related to cardio-vascular diseases. Among these, both PhotoPlethysmoGraphy (PPG) and Electrocardiography (ECG) signals are those more employed. PPG signals are an emerging non-invasive measurement technique used to study blood volume pulsations through the detection and analysis of the back-scattered optical radiation coming from the skin. ECG is the process of recording the electrical activity of the heart over a period of time using electrodes placed on the skin. In the present paper we propose a physiological ECG/PPG “combo” pipeline using an innovative bio-inspired nonlinear system based on a reaction-diffusion mathematical model, implemented by means of the Cellular Neural Network (CNN) methodology, to filter PPG signal by assigning a recognition score to the waveforms in the time series. The resulting “clean” PPG signal exempts from distortion and artifacts is used to validate for diagnostic purpose an EGC signal simultaneously detected for a same patient. The multisite combo PPG-ECG system proposed in this work overpasses the limitations of the state of the art in this field providing a reliable system for assessing the above-mentioned physiological parameters and their monitoring over time for robust medical assessment. The proposed system has been validated and the results confirmed the robustness of the proposed approach.

## 1. Introduction

The monitoring of dynamic changes of physiological and biological parameters through a non-invasive integrated systems can play an important role in a wide variety of applications including healthcare and sports training fields [1,2,3]. Among these, hemodynamic parameters (e.g., heart rate, tissue perfusion) obtained through PhotoPlethysmoGraphy (PPG) measurements and heart electrical activity (ECG) are certainly investigated a lot due to their impact on cardiovascular diseases [4,5]. However, non-invasive integrated systems measuring these parameters require very clean physiological signals to allow robust and effective computation of the medical indicators. In this context we propose a very robust and efficient bio-inspired pattern recognition pipeline for PPG and ECG signal filtering. 

Before detailing the proposed pipeline, a brief introduction on PPG and ECG signals is provided to give an overview of the current processing art. After that, the hardware system used for the test and the proposed bio-inspired algorithm are described. Finally, the validation results are discussed to confirm the effectiveness of the adopted approach.

## 2. PPG Physiological Analysis and Recognition: Description and Prior Art

PhotoPlethysmoGraphy (PPG) is a signal obtained by a non-invasive low-cost optical technique which is becoming popular to provide information about the cardiovascular system. Several physiological parameters like heart pulse, respiratory rate, tissue perfusion and some vascular and cardiac disorders can be easily monitored via PPG. This makes PPG signals very appealing to assess vascular diseases, especially the effects of vascular aging, hypertension and atherosclerosis, providing also information on arterial stiffness and elasticity [3]. 

PPG can be also used to detect blood volume changes in the microvascular bed of human tissue [4,5]. Additionally, PPG is often employed in a non-invasive manner to make measurements at the skin surface. A PPG waveform comprises a pulsatile (“AC”) physiological waveform which can be attributed to cardiac-synchronous changes in the blood volume upon each heart-beat, superimposed on a slowly varying (“DC”) baseline with various lower frequency components which is attributed to respiration, thermoregulation, the nature of skin tissues, and so on. Actually, in each cardiac cycle the heart pumps blood to the periphery distending the arteries and arterioles in the subcutaneous tissue. 

**Figure 1:**
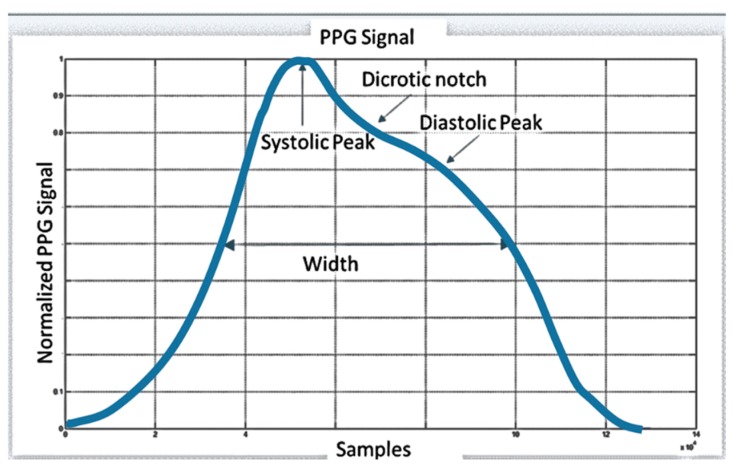
Compliant standard PPG waveform.

If a light detector device is attached over the skin, a pressure pulse from the venous plexus can be also detected as a small secondary peak. The change in volume caused by the pressure pulse can detected by illuminating the skin with light from a light-emitting diode (LED) and then by measuring the amount of light either transmitted or reflected to a photodiode [6]. Each cardiac cycle appears as a peak. Figure 1 shows a classical PPG compliant waveform.

The flow of the blood to the skin can be modulated by several physiological parameters, so that PPG can be also used to monitor breathing, hypovolemia and circulatory conditions and subjective analysis as well. Furthermore, the shape of the PPG waveform differs from subject to subject and varies as a function of the location of the pulse sensor [6].

PPG signals may be also useful in other areas. For instance, it has been considered to use them in the automotive field to gain information about the behavior and/or n the reactions of drivers and passengers in various situations which may occur in a motor vehicle [7,8]. Unfortunately, the physiological sampling pipeline presents some noise or signal artifacts (motion artifacts, electronic noises, signal distortion, sensors issue, random noises, etc.) with PPG waveforms even after a careful filtering of the raw signal. For this reason, a PPG compliant waveform recognition mechanism is needed to improve the robustness of the medical indicators computed from source PPG data [9].

In [9,10] the authors have proposed a reconstruction pipeline for PPG signals based on usage of the discrete wavelet transform (DWT) in combination of EMD methodology. In [11,12] a pipeline based on usage of step-size and adaptive Least Mean Squares (LMS) filters for removing motion artifacts in PPG signals is described. In [10] the authors provided a full PPG signal analysis pipeline for Pulse Wave Velocity (PWV) measurements as medical indicators for cardiovascular risk assessment. In [13] an interesting approach for motion artifact reduction was proposed. The main core of the proposed method is to properly combine some features of the PPG (the quasi-periodicity of the PPG signal) with the absence of correlation between the same signal and the motion artifact patterns. The pipeline completed the showed analysis with an independent component analysis step followed by ad-hoc low-pass filtering providing a full pipeline for a robust PPG signal filtering and compliant waveform recognition. Anyway, the various approaches in processing PPG signals may include using, basically, conventional digital filters (FIR/IIR), conventional DTW (Dynamic Time Warping), heuristic algorithms; neural networks and fuzzy systems, deep and machine learning methods. These approaches may result in a PPG signal processing pipeline having various limitations such as:

- high complexity of the system, which may result in a long computational time, which is hardly compatible with time constraints applicable to medical devices;
- a modest sensitivity/specificity ratio v. high computational costs;
- arrangements based on neural networks or fuzzy systems involve training sessions (e.g., in connection with over-fitting issues, neural network topology, training algorithms, etc.) or self-tuning of adaptive parameters;
- certain arrangements (irrespective of whether software-based or hardware-based) are not easy to implement.

The proposed pipeline has been developed for addressing the above drawbacks as confirmed in the validation section of this article.

## 3. The PPG Detection System

**Figure 2:**
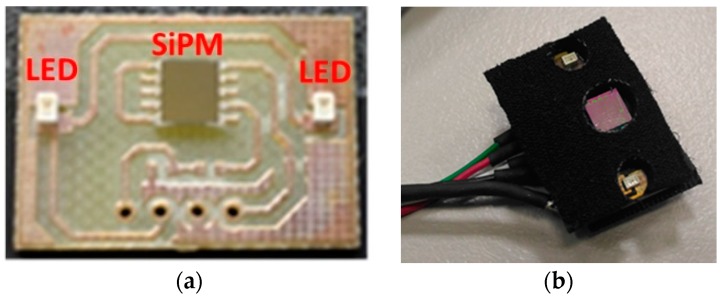
PPG probe comprising a SiPM detector and LED emitters. Detailed PCB (**a**) and the package (**b**) LT M673 LEDs (OSRAM, Milan, Italy) based on InGaN technology (in SMD package) emitting at 529 nm and have been used as optical light sources [16]. The LEDs have an area of 2.3 × 1.5 mm^2^, viewing angle of 120°, spectral bandwidth of 33 nm and typical power emission of a few mW in the standard operation range.

The PPG probe used in this study is shown in Figure 2. It comprises two main components: (a) silicon photomultipliers (SiPMs) detector and (b) LED photon emitters:

(a) The SiPM detector is fabricated by STMicroelectronics (Catania, Italy) [14] and features a total area of 4.0 × 4.5 mm^2^ and 4871 square microcells with 60 µm of pitch. It has a geometrical fill factor of 67.4% and is packaged in a surface mount housing (SMD) of 5.1 × 5.1 mm^2^ total area [15,16]. A Pixelteq dichroic bandpass filter(Bryan Dairy Rd, Largo, FL, USA) centered at 542 nm (Full Width at Half Maximum (FWHM) of 70 nm and optical transmission higher than 90% in the pass band range) was glued on the SMD package by using 352TM adhesive (Loctite^®^, Milan, Italy). In this configuration, in the driving range 0–3 V the device has a maximum Photon Detection Efficiency (PDE) of about 29.4% at 565 nm and of about 27.4% at 540 nm (central wavelength in the filter pass band). Furthermore, it has been proved that the dichroic filter reduces the absorption of environmental light of more than 60% in the linear operation range of the detector operating in Geiger mode above its breakdown voltage (~27 V).

**Figure 3:**
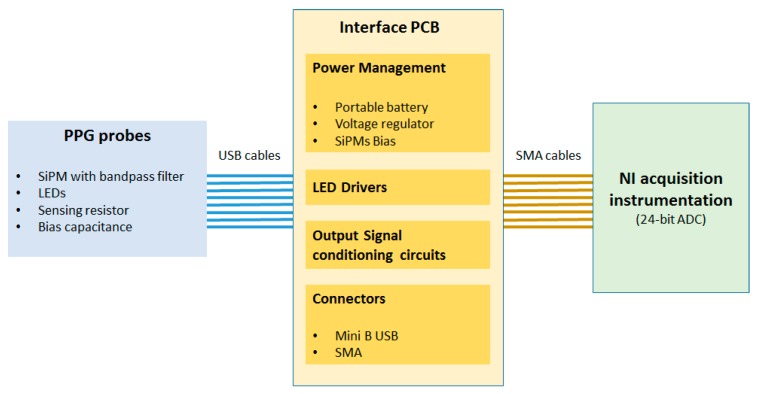
PPG Signal Sampling pipeline: System Architecture.

The block scheme of the PPG probe system architecture is reported in Figure 3. A specific printed circuit board (PCB) was developed to interface the PPG probe with a NI (National Instruments, Austin, TX, USA) acquisition instrumentation used to measure the PPG signals. The PCB is provided by 4 V portable battery, power management circuits, conditioning circuit for the output SiPMs signals, eight mini B-USB connectors for PPG probes and eight SMA output connectors. The device was power supplied at 3.3 V by a specific voltage regulator. In the same board a step-up DC-DC converter generates an output of 30 V and provides a bias to the SiPMs. Trimmers on the PCB allow adjustment of the LEDs brightness of PPG probe. The continuous component of the output SiPMs signals was partially eliminated using a differential signal acquisition configuration. The subtraction of the continuous part is adjustable by the trimmer to optimize the output signal acquisition in each ADC channel.

A LabVIEW software program was developed to acquire the PPG signals. The software controls the 24 bit ADC NI PXle-4303 NI acquisition system and includes a graphical user interface (GUI) allowing to display the filtered PPG signal, its first and second derivatives. It is also possible to compare two PPG signals acquired from probes arranged at different body-sites and measure their temporal delay. During the acquisitions the working sampling frequency was set to 1 kHz. Finally, the overall dataset is stored in a log file that can be selected by the user and handled by a MATLAB^®^- based algorithm for PPG signal pattern recognition. 

**Figure 4:**
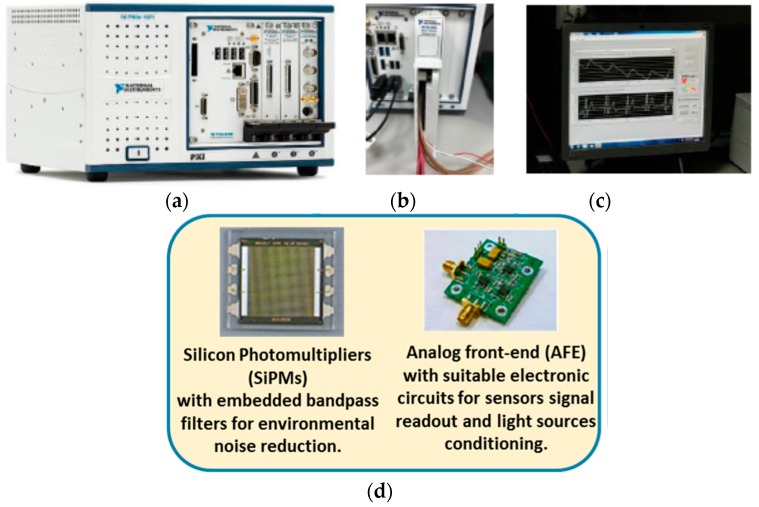
PPG probe hardware overview. (**a**) National Instrument (NI) device with acquisition board (containing 24 bit ADCs). Detailed ADC/DAC acquisition system of the NI device (**b**). NI system output during the acquisition of PPG and ECG signals (**c**). Such output is obtained by means of a software developed by NI in a LabView environment. Details of the SiPM sensor and electronic circuits for PPG signal filtering and transduction (**d**).

In order to dump the environmental light and improve the signal to noise ratio, SiPM was equipped with an embedded optical filter. Measurements have been conducted in reflectance mode on the right radial artery. Figure 4 shows an overview of the PPG probe hardware used for the proposed pipeline [17].

**Figure 5:**
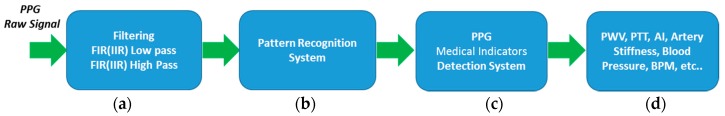
PPG Pattern Recognition Pipeline. PPG raw signal filter (**a**), the PPG pattern recognition system (**b**), medical indicators detection system (**c**) and indicators extraction stage (**d**).

Figure 5 reports the block-scheme of the processing pipeline applied to collected PPG signals. It is composed by four blocks: (a) PPG raw signal filter (Figure 5a), (b) the PPG pattern recognition system (Figure 5b), (c) medical indicators detection system (Figure 5c) and (d) indicators extraction stage (such as pulse wave velocity (PWV), pulse transit time (PTT), cardiovascular assessment stage (A1), artery stiffness, blood pressure measurement/monitoring, beats per minute (BPM) and so on) (Figure 5d) [18,19].

The first block (PPG raw signal filter) operates a PPG signal filtering. PPG raw signals are digitally filtered by using a finite impulse response (FIR) filter [17] available in the MATLAB^®^ specific tool Filter Design & Analysis Tool. In our specific case, a set of FIR filters are configured to work as a low/high-pass and filters for filtering at the range of 1–10 Hz, allowing to remove the 50 Hz power line frequency noise and other signal artifact as well. Suitable FIR coefficients were obtained by using the EquiRipple [15] method which allows to avoid any phase distortion issues. Table 1 shows the configuration parameters of the used FIR filters.

**Figure 6:**
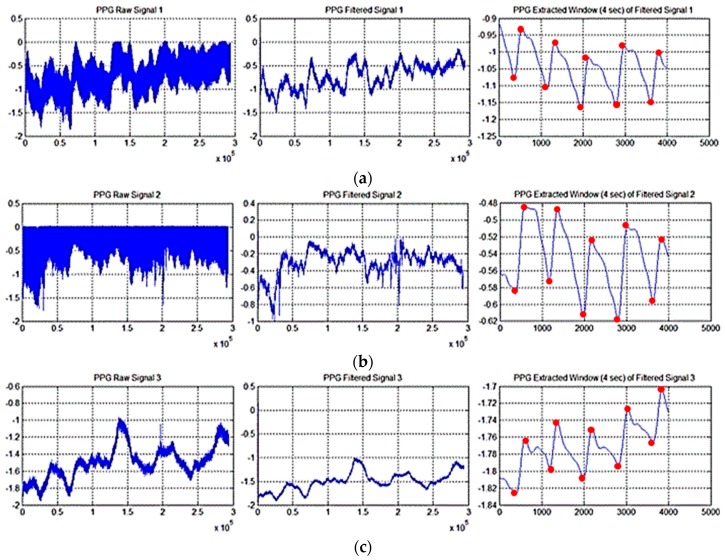
(**a**) The plot in the first column reports the PPG Raw Signal 1 (left wrist). The second window reports same PPG raw signal filtered as per FIR filters previously described. Latest column shows an enlarged window of the filtered PPG signals; (**b**) The plot in the first column reports the PPG Raw Signal 2 (right wrist). The second window reports same PPG raw signal filtered as per FIR filters previously described. Latest column shows an enlarged window of the filtered PPG signals; (**c**) The plot in the first column reports the PPG Raw Signal 3 (ankle). The second window reports same PPG raw signal filtered as per FIR filters previously described. Latest column shows an enlarged window of the filtered PPG signals.

Figure 6 reports three sampled PPG signals: the raw signal (labeled as 1), the filtered signal (labeld as 2) and the enlarged time-window of the FIR-filtered signal (labeled as 3). The second block is the PPG Pattern Recognition System (PRS). The waveform of the PPG signals (Figure 1) is featured by a number of parameters like the width, the systolic peak, dicrotic notch and the diastolic peak. It is known from literature that the standard PPG waveform shows some differences in its pattern depending on the body-site (ears, fingers, toes) in which the signal is measured [10,17].

The complete filtering of the PPG waveforms has been carried out using a fully automatic PRS block. The PRS algorithm has been developed as MATLAB^®^ scripts and functions and is composed by two main steps:

- FDA: First derivative analysis for the assessment of the PPG pulse characteristics.
- PRW: Pattern recognition of PPG waveforms.

**Figure 7:**
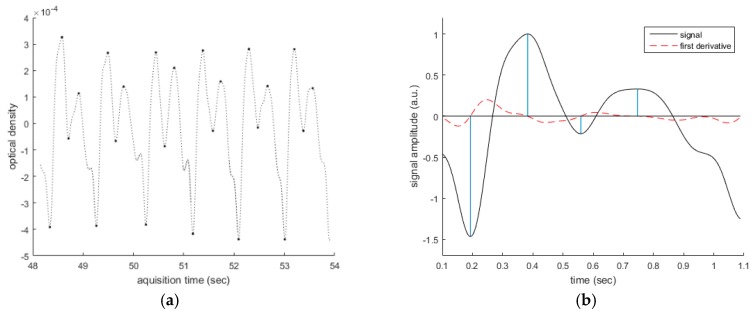
Representative results of FD Analysis (**b**) of the PPG signal (**a**). The analysis of the FD of the PPG signal allows the detection of the maximum and minimum points of the timeseries.

The FDA step identifies the extremes by considering the first derivative (FD) of the PPG timeseries. A careful analysis of the FD of the PPG signal allows the detection of the maximum and minimum points of the whole PhotoPlethysmoGraphy timeseries. We suppose to find a PPG waveform between two subsequent local minima so that we segment each pattern identifying the minimum points of the analyzed timeseries. Moreover, extended first and second derivative analysis is suitable to find maximum and dicrotic points of each so segmented PPG pattern. Figure 7 shows a representative result of the FD Analysis of the filtered PPG timeseries.

After that, a processing of each segmented PPG waveform is carried out in order to discriminate if it is compliant or not. The main core of this step is based on the use of bio-inspired nonlinear dynamic mathematical models reported by the following equations [17,20]:

(1) $$\begin{array}{c} \begin{array}{c} \frac{\partial x_{1}}{\partial t}=-x_{1}+\left(1+\mu \right)y_{1}-\beta y_{2}+\rho_{1}\\ \frac{\partial x_{2}}{\partial t}=-x_{2}+\left(1+\mu \right)y_{2}+\beta y_{1}+\rho_{2} \end{array}\\ y_{j}=\frac{1}{2}\left(\left|x_{j}+1\right|-\left|x_{j}-1\right|\right);\;j=1,2 \end{array}$$

**Figure 8:**
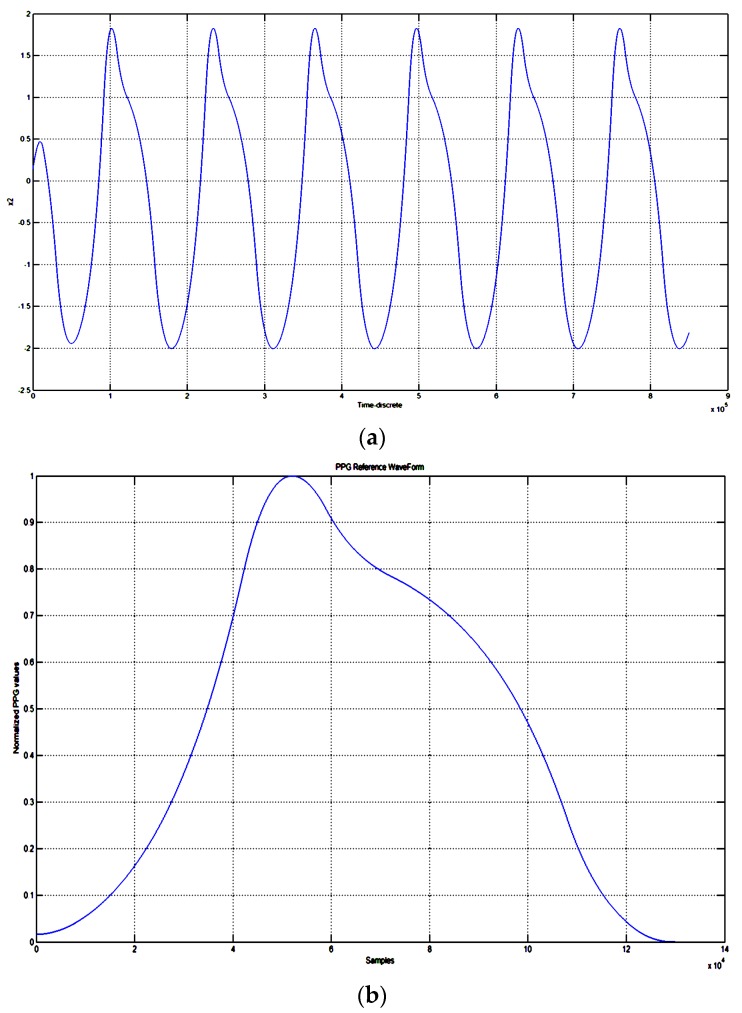
Dynamic evolution of the *x*_2_ variable (**a**). Detail of the normalized [0, 1] single waveform of the *x*_2_ time-evolution (**b**).

Equation (1) shows a typical nonlinear biological reaction-diffusion (RD) system suited for managing locomotion in bio-inspired robots. Each variable (*x*_1_ and *x*_2_) represents the so-called moto-neurons so that the evolution of each variable shows a biological dynamic as involved in locomotion of such biological species. The set of the system parameters was found to be suitable for defining a specific type of nonlinear dynamics (specifically: *x*_2_ variable) for the modelled RD process, which is close to a standard PPG timeseries. Figure 8 shows the steady-state autonomous oscillations of the variable *x*_2_ with detail of single waveform. The parameters used in Equation (1) are the following [20]:

$$\mu =0.5;\;\rho_{1}=-0.3;\;\rho_{2}=0.3;\;\beta =1;\;x_{1}\left(0\right)=0.1;\;x_{2}\left(0\right)=0.08$$

Considering the single waveform of *x*_2_ dynamics, after a normalization in the interval [0, 1] and resizing e.g., by means of the method proposed in [21], a pattern was found to be well adapted and used as PPG reference wave generator for a pattern recognition system (PRS).

The above reported nonlinear mathematical model (1) can be implemented by means of the Cellular Neural Network (CNN) methodology [20]. These are an analogic time-continuous space-discrete grid of neural cells properly coupled [22,23]. Due to its analogic implementation, the CNNs are able to perform such operations with high-speed computational capability i.e., near real-time [22,23]. The Equation (2) shows the mathematical model of a MxN CNNs (specifically for the so-called State Controlled CNNs [24] which is an extended version of the original CNNs proposed by Chua and Yang in [25]):

(2) $$\begin{array}{ll} C\frac{dx_{ij}\left(t\right)}{dt}=-\frac{1}{R_{x}} & x_{ij}\\ & +\sum_{C\left(k,l\right)\in N_{r}\left(i,j\right)}A\left(i,j;k,l\right)y_{kl}\left(t\right)+\sum_{C\left(k,l\right)\in N_{r}\left(i,j\right)}B\left(i,j;k,l\right)u_{kl}\left(t\right)\\ & +\sum_{C\left(k,l\right)\in N_{r}\left(i,j\right)}C\left(i,j;k,l\right)x_{kl}\left(t\right)+I \end{array}$$

where:

$$\left(1\leq i\leq M,1\leq j\leq N\right)\;y_{ij}\left(t\right)=\frac{1}{2}\left(\left|x_{ij}\left(t\right)+1\right|-\left|x_{ij}\left(t\right)-1\right|\right)\;N_{r}\left(i,j\right)=\left\{C_{r}\left(k,l\right);\left(max\left(\left|k-i\right|,\left|i-j\right|\right)\leq r,1\leq k\leq M,1\leq l\leq N\right)\right\}$$

In Equation (2) *x_ij_*(*t*) represents the state of the single cell *C(i,j)*, while *y_ij_*(*t*) and *u_ij_*(*t*) represents the output and the input of the cell *C*(*i*,*j*). CNNs are arrays of nonlinear and simple computing elements characterized by local interactions between cells. The dynamic of a CNNs cell *C*(*i*,*j*) with electronic circuit [20,25] is described by Equation (2) in which both the input and the output of the neighbourhood coupled cells are represented by the voltages values *u_kl_* and *y_kl_*. The neighbourhood of single cell *C*(*i*,*j*) is mathematically represented by *N_r_*(*i*,*j*) while the type of cell-coupling is defined by the elements of the so-called cloning matrix templates *A*(*i*,*j*;*k*,*l*), *B*(*i*,*j*;*k*,*l*), *C*(*i*,*j*;*k*,*l*) as well as by the bias *I*.

A CNN paradigm is thus well suited to describe locally interconnected simple dynamical systems showing a lattice-like structure. CNNs are conventionally used for various types of applications such as image and signal processing, bio-inspired system modelling, or high-speed resolution of partial differential equations (PDEs) [26,27]. They are particularly employed when the emulation of PDEs solutions involves the evolution of each variable over time, its position (in space) and its interactions deriving from the space-distributed structure of the whole system (indeed, the numerical solution of PDEs almost inevitably involves spatial discretization). CNN paradigm represents a helpful tool in the real-time simulation of spatio-temporal phenomena. In the case of the reaction-diffusion model PDEs herein considered, RD CNNs are used [26,27].

The PPG reference waveform generated by (1) implemented through CNNs (2) is normalized and rescaled to be time-comparable with acquired segmented pre-filtered PPG waveform. The system performs a rescaling of the previously acquired segmented and pre-filtered PPG waveform with the aim to get it time-comparable with PPG reference waveform. In order to keep high speed computation of the pipeline, the rescaling algorithm is based on CNNs as described in [28]. The two PPG waveforms, *p*_1_(*k*) acquired and *p*_2_(*k*) reference (both resampled as per *N_s_* number of rescaled samples), are normalized into [0, 1]. Finally, an ad-hoc normalized sample cross-correlation analysis $\rho_{p_{1}p_{2}}\left(h\right)$ is performed (see Equation (3)) in order to have a compliance measure for the analyzed PPG waveform [29]:

(3) $$\gamma_{p_{1}p_{2}}\left(h\right)=\frac{1}{N_{s}}\overset{N_{s}-h}{\underset{k=1}{\sum }}\left(p_{1}\left(k\right)-\mu_{p_{1}}\right)\left(p_{2}\left(k+h\right)-\mu_{p_{2}}\right)\;h=0,1,2\;\sigma_{p_{1}p_{1}}=\sqrt{\gamma_{p_{1}p_{1}}\left(0\right)};\;\sigma_{p_{2}p_{2}}=\sqrt{\gamma_{p_{2}p_{2}}\left(0\right)}\;\mu_{p_{1}}=\frac{1}{N_{c_{1}}}\overset{N_{c_{1}}}{\underset{j=1}{\sum }}p_{1}\left(j\right);\;\mu_{p_{2}}=\frac{1}{N_{c}{}_{2}}\overset{N_{c_{2}}}{\underset{j=1}{\sum }}p_{2}\left(j\right)\;\;\rho_{p_{1}p_{2}}\left(h\right)=\frac{\gamma_{p_{1}p_{2}}\left(h\right)}{\sigma_{p_{1}p_{1}}\sigma_{p_{2}p_{2}}}\;h=0,1,2$$

**Figure 9:**
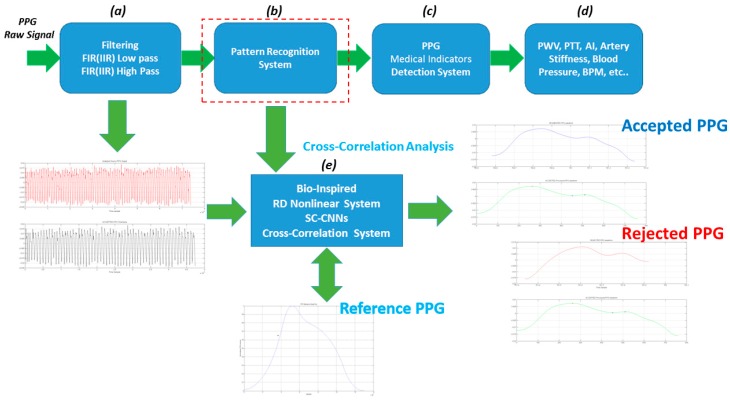
The pipeline is composed by the PPG raw signal filter, consisting of a FIR pass-band scheme (**a**). The second block is the PPG pattern recognition system (**b**), which is followed by the medical indicators detection system (**c**) and the indicators extraction stage (**d**). The Figure also shows a schematic illustration of how the proposed Pattern Recognition System (PRS) works (**e**).

**Figure 10:**
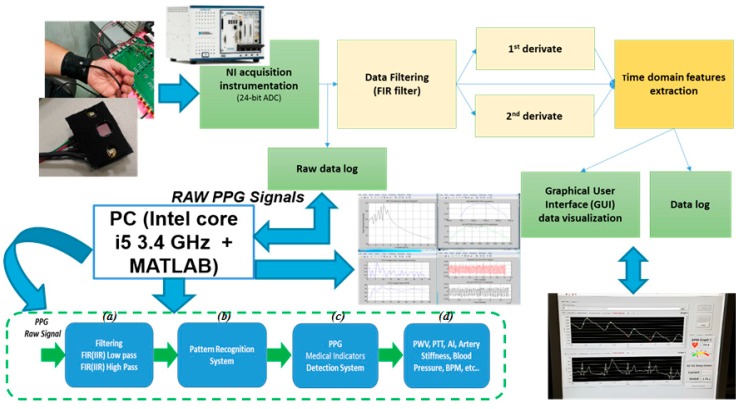
Detailed description of the whole PPG Pipeline (blocks (**a**–**d**)) with description of hw pipeline developed for signal acquisition with NI device (Data filtering, derivative analysis of acquired PPG signal, GUI and data visualization stage of the acquired physiological data).

Only the PPG patterns showing in average high normalized sample cross-correlation (≥0.90) are considered to be compliant, while the other ones are discarded. The results confirm the robustness and effectiveness of the approach herein described showing very promising sensitivity/specificity higher than 97%. It is important to underline that we are proposing a mathematical analytic nonlinear model for generating PPG waveform. This model includes adaptive parameters suitable to be changed to provide different PPG patterns according to the body-measure site such as ear, thumb, toe, and so on. In this way, a very efficient and robust pattern recognition system self-adapting to the PPG measure site is achieved. Figure 9 shows the details of the PRS Block. As a summary, Figure 10 reports the scheme of the whole PPG Pattern Recognition Pipeline architecture.

## 4. EEG Physiological Analysis and Recognition

**Figure 11:**
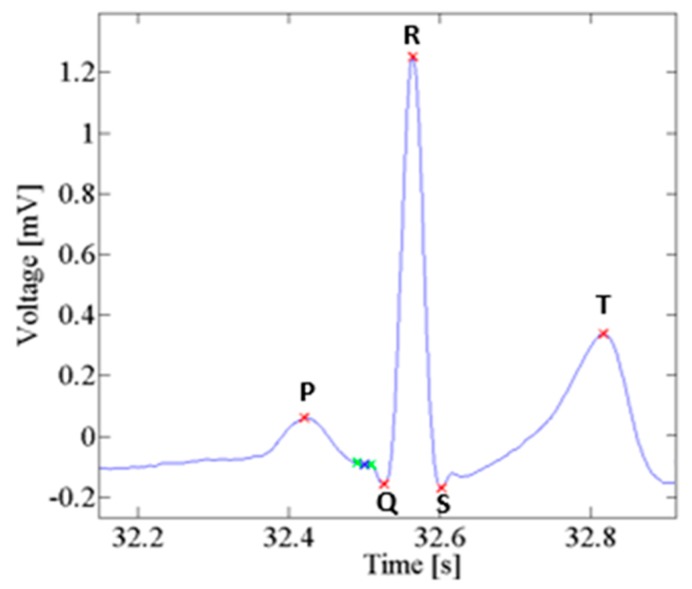
A compliant EEG standard waveform.

Electrocardiography (ECG) is the process of recording the electrical activity of the heart over a period of time using electrodes placed on the skin. These electrodes detect the tiny electrical changes on the skin arising from electrophysiological patterns of de-polarization and re-polarization which occurs during each heartbeat of the heart muscle. Electrocardiography is a cardiology test commonly performed. A typical ECG waveform includes two intervals: (a) PR interval including both P waveform and PR segment; (b) the QT interval including Q, R, S waveforms (QRS complex), ST segment and T waveform, respectively [30]. Figure 11 shows a classic ECG waveform.

**Figure 12:**
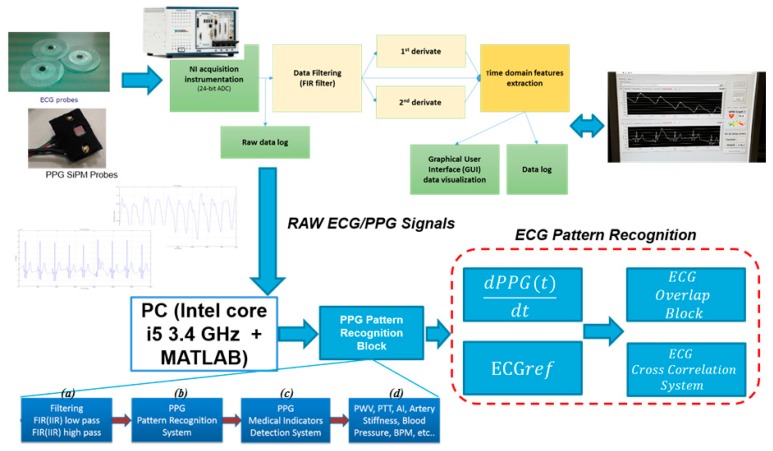
Overall System Architecture of the PPG/ECG combo system with Patter Recognition Pipelines. The figure shows detailed about PPG recognition stage (blocks (**a**–**d**)) and ECG PR blocks (ECG Pattern Recognition).

Detecting and processing ECG signals is the subject matter of extensive literature. ECG signal sampling is often affected by different type of signal corruptions such as artifacts due to motion or micro-movements of the patient body, electronic noise, sensor issues, etc. In [30] the authors propose a time-domain based approach resulting of combination between a classical dynamic time warping with classical ECG indicators, such as heart-rate and amplitude. In [31] a method based on the use of Artificial Neural Networks ANN (Self Organizing Map, Back Propagation MLP, Learning Vector Quantization) is shown as robust approach for ECG pattern recognition. In [32] an interesting approach for ECG pattern recognition based on model of syntactic/linguistic representation of ECG waveform is described. An alternative method for ECG classification and recognition is reported in [33] and it is based on the use of frequencies spectrogram with ad-hoc features. In [34] the authors successfully use the concept of isoelectric curve and a fuzzy clustering for detecting correct ECG waveform in a continuous recorded time-series, while in [35] a learning algorithm based on computation of the so called gray relational coefficient for ECG waveform classification is proposed. In [36,37] the recent approaches based on Deep Learning Algorithm and Convolutional Neural Networks have been used. In [38] an interesting approach for ECG signal processing was introduced based on the use of the so called Empirical Mode Decomposition (EMD) originally applied for respiratory signal evaluation. EMD is a method useful to study nonlinear features of a signal or a time series. EMD allows the signal or a time-series to be separated into intrinsic oscillations using local temporal and structural data features. This approach can be effective applied to replace traditional methods (Fourier analysis, wavelet transform, etc.) for analyzing signals, specifically, physiological time series such as the ECG. The authors describe an efficient method for ECG pattern recognition and filtering based on the use of the corresponding PPG signal sampled in the “combo” PPG/ECG system. The ECG signal sampling is made by using classical electrical sensor-probes placed in the patient body as per standard leads configuration. In our validation setup a standard Einthoven triangle leads configuration for acquiring a robust ECG signal is employed [39,40]. Figure 12 shows the system architecture of the “combo” hardware platform used for sampling EEG and PPG signals. 

**Figure 13:**
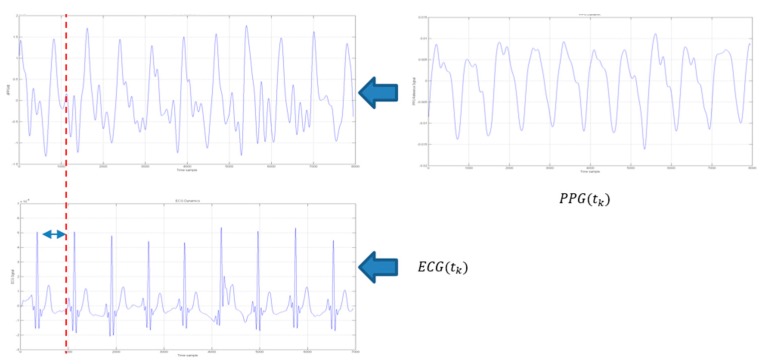
The *PPG*(*t_k_*) to *ECG*(*t_k_*) correlation diagram. The variable *t_k_* represent a time-onset of the segmented PPG/ECG waveforms. The first window on the left, reports the PPG first derivative ($\left(dPPG\left(t_{k}\right)/dt\right)$ in the time-onset *t_k_*.

A clean PPG signal is presented and/or exploited in conjunction with the ECG signal to understand if the ECG signal being found consistent (compliant) with the PPG signal. To some extent such approach can be regarded as alternative or complementary to ballistocardiography (BCG). BCG is a device able to measure ballistic forces on the heart producing a graphical representation of repetitive motions of the human body arising from the sudden ejection of blood into the great vessels at each heartbeat. It is a vital sign in the 1–20 Hz frequency range caused by the mechanical movement of the heart. It can be recorded by noninvasive methods from the surface of the body. The approach herein proposed is based on the recognition that, as occurred in BCG analysis, an observable cross-correlation exists between the first-derivative of a PPG processed waveform and ECG signal for a same patient. Figure 13 depicts such correlation.

The processing system herein proposed includes a number of processing modules/circuits (Figure 12) that are:

- a block “*ECG_ref_*(*t_k_*)” configured for making available an ECG reference signal (i.e., a conventional ECG standard pattern stored in the ECG/PPG system or possibly loaded on-demand);
- a block “*dPPG*(*t_k_*)/*dt*” configured for calculating a first-derivative PPG waveform for use in analyzing the related ECG waveform;
- a block “ECG Overlap Block” configured for calculating a degree of cross-correlation of the first-derivative PPG waveform and the related ECG waveform;
- a block “ECG Cross Correlation System” configured for calculating a degree of cross-correlation between the ECG reference signal waveform with the (detected) ECG waveforms to be analyzed.

A validation block, which can be merely exemplified as a logical AND gate, is sensitive to the outputs from the previous two blocks analyzing certain cross-correlation threshold values to understand if the analyzed ECG waveform is compliant or not. 

When the outputs from the blocks “ECG Overlap Block” and “ECG Cross Correlation System” reach certain cross-correlations threshold values, they are indicative of the quality of the sampled ECG waveforms if they are adequate to be valid and reliable to be used for diagnostic purposes by a practitioner. This is the output result of validation signal coming from the validation block. The approach herein presented relies on a sort of “double check” involving both first-derivative PPG cross-correlation block (block “ECG Overlap Block”) and ECG standard pattern cross-correlation (block “ECG Cross Correlation System”) analysis which facilitates a high degree of reliability. The PPG/ECG pattern recognition system will be described in details in the following paragraphs.

**Figure 14:**
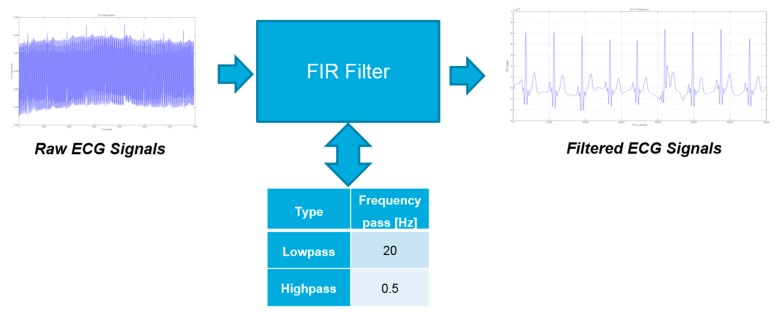
The ECG filter system (FIR).

Preliminary, a band-pass filtering is applied to the sampled ECG signal (Figure 14). This filtering is similar to that applied to the PPG signal (e.g., a low-pass section and high-pass section) except for the different choice of the cut-off frequencies (e.g., 0.5 Hz and 20 Hz respectively for high-pass and low-pass for the ECG signal).

The system architecture diagram (Figure 12) includes a processing of the ECG signals and the PPG derivative signal so that the compliant first-derivative PPG waveform can be used to analyze the related ECG waveform obtained by automatic segmentation of pre-filtered ECG from the in the same PPG time onset. For that purpose, first-derivative PPG and the ECG waveforms can be normalized over the interval [0, 1]. Time-rescaling and shifting are performed [21] in order to time-align the peaks of the various signals involved:

- the ECG waveforms and the first-derivative PPG
- the ECG waveforms and the ECG reference waveform

In this way, cross-correlation analysis of these signals can be facilitated by relying on time alignment (overlap) of the respective peaks.

**Figure 15:**
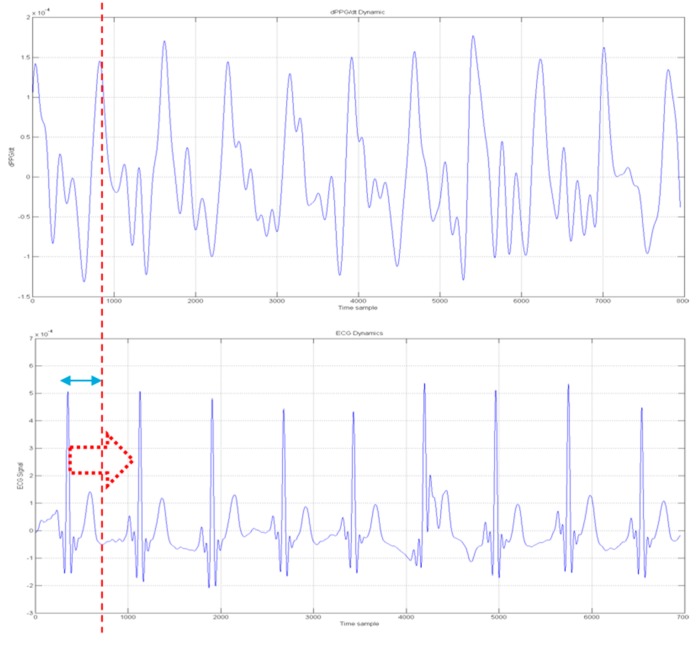
ECG(t_k_) (bottom) to $\mathrm{dPPG}\left(t_{k}\right)/\mathrm{dt}$ (top) analysis.

Figure 15 shows the ECG(t_k_) to dPPG(t_k_)/dt analysis as reported in the following equation:

(4) $$ECG^{j}\left(t_{k}\right)->ECG^{j}\left(t_{k}+\delta_{k}^{j}\right)\;\forall j=1..N_{ECG};\;\forall \;t_{k}$$

where t_k_ indicates the PPG time-onset while δ_k_^j^ indicates the offset needed to align the dPPG(t_k_)/dt peak with ECG(t_k_) ones. The variable *N_ECG_* indicates the number of segmented ECG patterns. 

**Figure 16:**
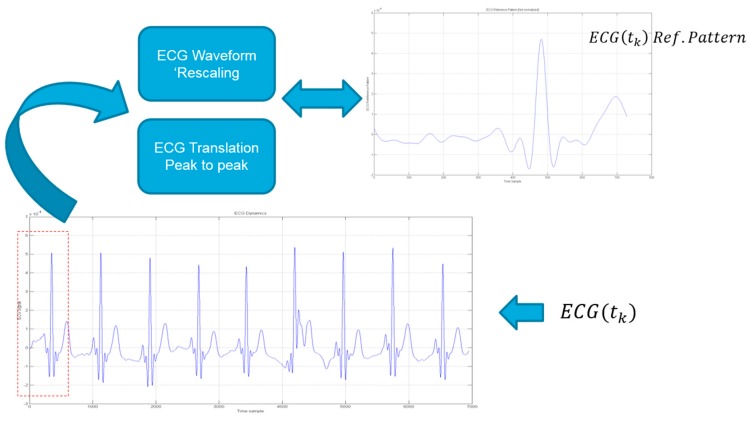
The ECG(t_k_) to ECG_ref_(t_k_) analysis: t_k_ indicate the PPG/ECG time-onset.

Figure 16 shows the cross-correlation analysis with reference ECG(t_k_) waveform (ECG_ref_(t_k_) block).

For all ECG(t_k_) segmented waveforms (N_ECG_), the alignment between ECG_ref_(t_k_) peak with ECG(t_k_) ones is obtained with same signal re-mapping as per Equation (4).

Finally, a standard sample cross-correlation analysis is performed to generate respective cross-correlation scores (indexes) e.g., between rescaled-normalized ECG waveforms and first-derivative PPG waveforms and a standard ECG reference pattern. The obtained scores are compared with reference cross-correlation thresholds [29]. 

**Figure 17:**
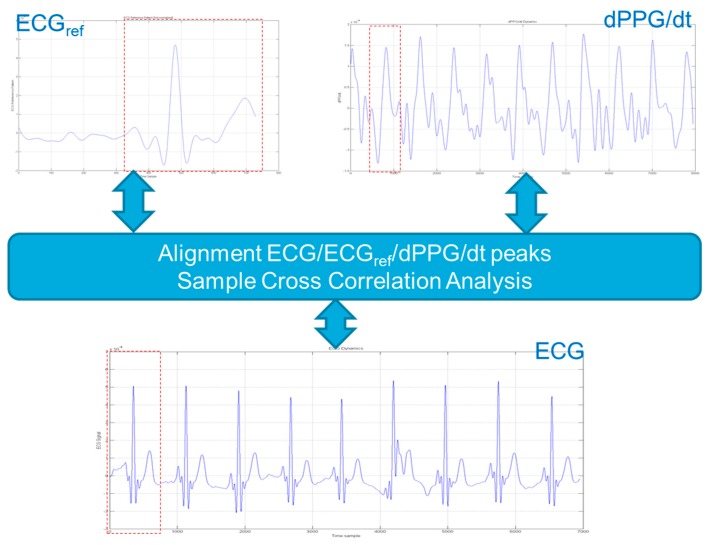
The system overview of the ECG pattern recognition pipeline based on sample cross-correlation of analyzed ECG waveform and ECG_ref_ and dPPG/dt pattern.

The whole proposed pipeline can be summarized as follows:

- “translating” (shifting in time) the sampled ECG waveforms to be analysed by causing their peaks (maxima) to correspond with the peaks in the first-derivative PPG signal and the peak of the ECG reference signal
- calculating (e.g., on signals normalized over the interval [0, 1]) sample cross-correlations between these signals, that is between:
- the sampled ECG waveforms and the first-derivative PPG signal;
- the sampled ECG waveforms and the ECG reference signal;
- comparing the sample cross-correlation indexes or scores with established compliance thresholds (values of 0.80 were found to represent a reasonable choice for both thresholds);
- the analysed ECG patterns having a sample cross-correlation indexes or scores reaching these thresholds (e.g., a cross-correlation equal to 0.80 or higher in both checks i.e., first-derivative PPG and ECG standard, respectively) will be considered a “conforming” ECG pattern to be retained; otherwise they will be discarded (Figure 17).
- validation is “ok” if both thresholds are reached so that ECG waveforms showing high cross-correlation with PPG-derivative waveforms and ECG reference waveform are “validated”, e.g., for diagnostic purposes.
- ECG waveforms showing low correlation with either one of the PPG-derivative waveform or the ECG reference waveform are discarded so that only “compliant” collected ECG waveforms can be used as a reference pattern for subsequent ECG analysis.

Even if this “double check” of the ECG signal is not mandatory per se, however this was found to facilitate the providing of reliable results with the former check (correlation with PPG-derivative) providing validation “as to form” and the latter check (correlation with ECG reference) providing validation “as to value/content”. 

**Figure 18:**
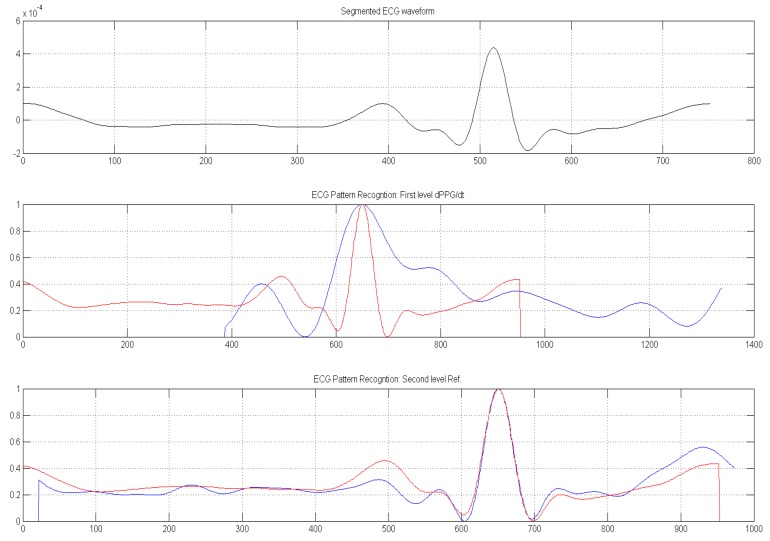
The ECG Patter Recognition GUI: First window shows the segmented ECG waveform according to segmented PPG onset (t_k_); The second ones reports segmented ECG (red) and first derivative of corresponding PPG waveform (dPPG/dt—blue) while the third window reports graphical representation of the segmented ECG (red) and reference ECG pattern (blue). Both waveforms plotted in second and third windows will be used for sample cross correlation analysis as previous described in Equations (3) and (4).

**Figure 19:**
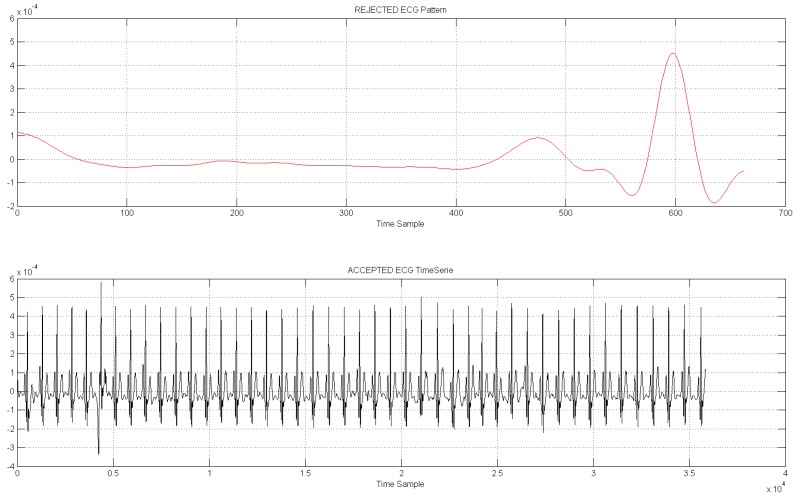
The accepted ECG time-serie (black signal in second window). The first window shows a sample of ECG pattern discarded by the proposed pattern recognition pipeline.

Figure 18 shows a diagram with the GUI reporting the pipeline useful for performing automatically ECG pattern recognition. Figure 19 shows an instance of a sampling accepted ECG time-serie filtered by the proposed pattern recognition algorithm.

## 5. Testing and Validation of the Proposed Method

**Figure 20:**
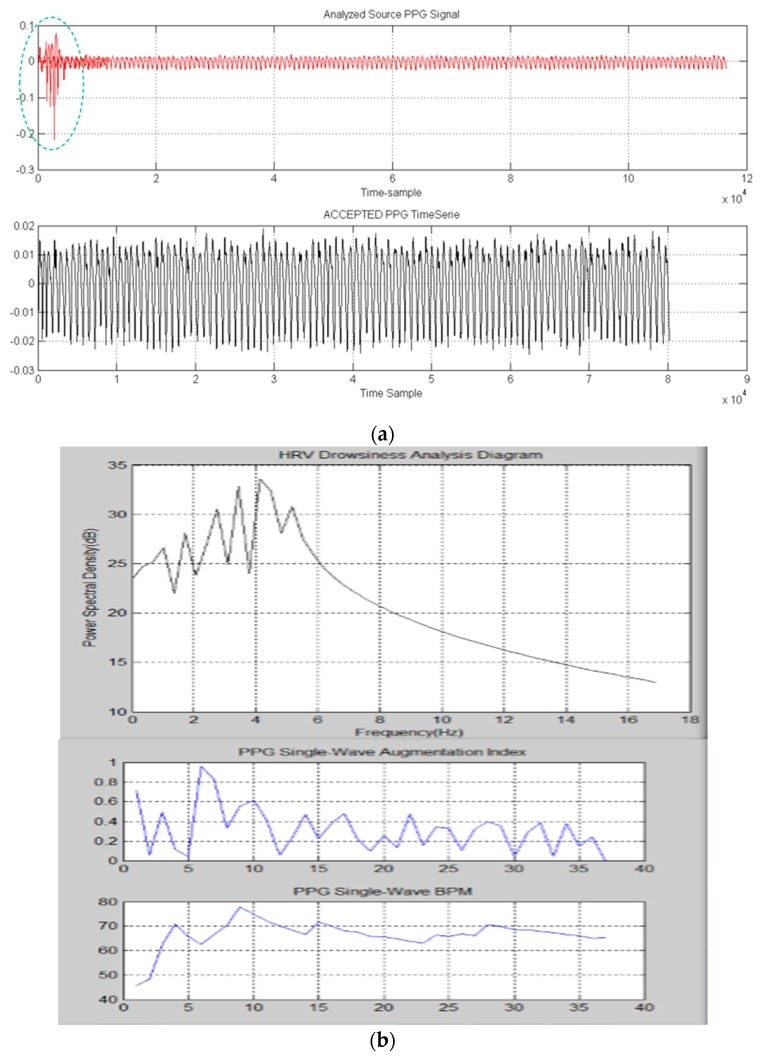

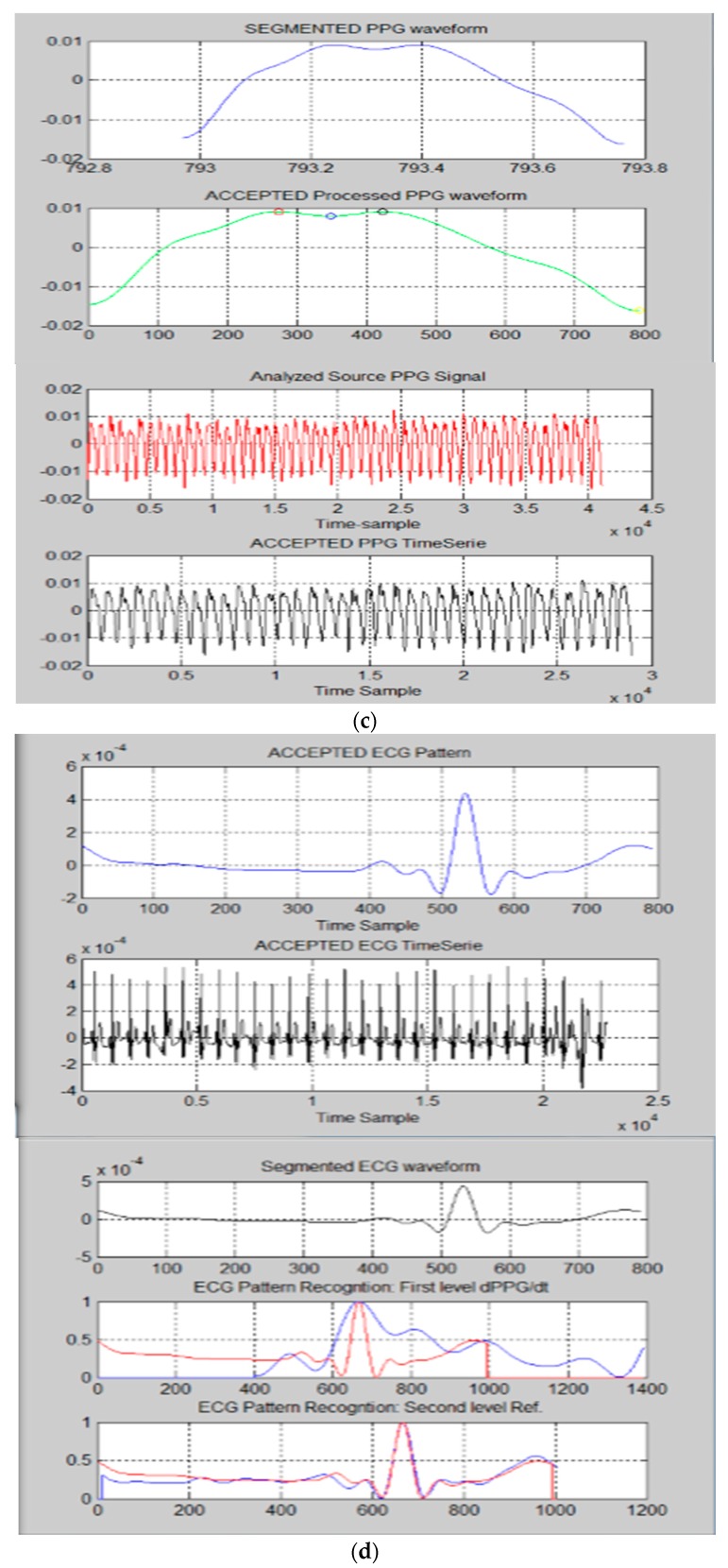
(**a**) The first sub-window shows an instance of noised PPG signal (red) properly filtered as reported in the second sub-window; (**b**) Medical indicators computation (HRV, Augmentation index (AI), BPM) included in the Pattern Recognition System GUI; (**c**) The described PPG pattern recognition system with detail about accepted PPG waveforms; (**d**) The proposed ECG pattern recognition diagrams with detail about accepted ECG waveforms.

The proposed pipeline has been tested and validated by using the SiPM based sensor hardware with LabView for sampling PPG signals (Figure 10). The collected PPG raw data were stored in a PC with INTEL i5 core 3.4 GHz CPU equipped with MATLAB^®^ and handled by the proposed PRS above described. The results are shown in the PC monitor with related medical indicators graphics such as the augmentation index (for artery stiffness), the BPM, etc. [18,19]. Figure 20 reports representative results for the robustness of the proposed pattern recognition algorithm as well as the developed MATLAB^®^ GUI. 

**Figure 21:**
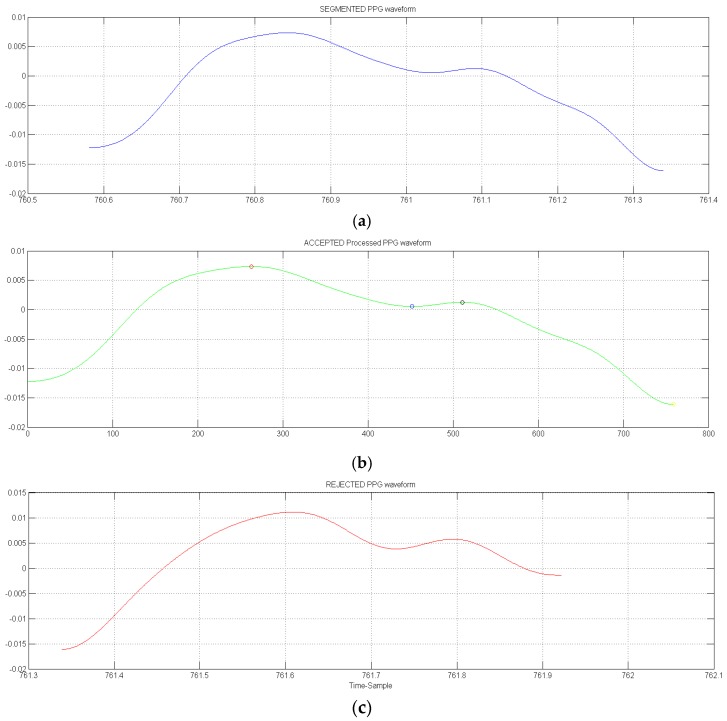
Representative results of accepted (blue PPG in (**a**)) vs rejected (red PPG in (**c**)) segmented PPG waveform. (**b**) shows accepted PPG waveform in which the described FD analysis is performed in order to detect the maximum (systolic peak), dichrotic and diastolic points (green waveform with highlighted points as circle).

The graph in Figure 21 reports a representative result of the filtered and properly selected PPG waveform after application of the proposed pipeline. In particular, the blue pattern is the segmented result, green pattern is the accepted waveform and the red patter is a uncompliant waveform that is rejected.

**Figure 22:**
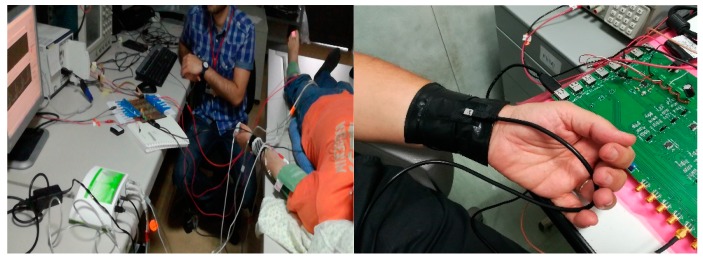
Some images of the validation setup used in our laboratories. In the first image we compare the processed PPG/ECG time-series with same ones obtained by commercial PPG/ECG medical device.

Figure 22 depicts some images of the experiments validated in our laboratory. The validation set was composed by 32 samples (10 min of PPG/ECG signals sampling for each ones) and fully confirmed the robustness of the proposed method with accuracy greater than 97% processing time near real time due to usage of the proposed bio-inspired model. The so processed PPG/ECG timeseries have been compared with same ones sampled in a classical commercial medical device. The medical indicators above mentioned (HRV, BPM, AI) have been computed in our PPG/ECG system and compared with same ones obtained with commercial devices confirming the robustness and effectiveness of the proposed approach. 

Future works aims on replacing the used signal rescaling algorithm with 1D version of the one proposed in [41] as preliminary results confirm an increasing of the specificity of the proposed approach. Moreover, in order to increase the robustness of the signal noise reduction integration of some steps of the noise reduction approach proposed in [42] are forecasted.

## 6. Patents

This proposed approach is included in the following IT Patent: Nr. 102017000081018 registered in July, 2017.

## Figures and Tables

**Table 1:** PPG filtering: FIR filter configuration parameters.

Type	Frequency Pass (Hz)	Frequency Stop (Hz)	Passband Attenuation (dB)	Stopband Attenuation (dB)
Low-pass	3.8	7.21	0.001	100
High-pass	1	0.3	0.01	40
